# Epidemiological patterns of cervical human papillomavirus infection among women presenting for cervical cancer screening in North-Eastern Nigeria

**DOI:** 10.1186/s13027-015-0035-8

**Published:** 2015-10-02

**Authors:** Mohammed Mohammed Manga, Adeola Fowotade, Yusuf Mohammed Abdullahi, Aliyu Usman El-nafaty, Danladi Bojude Adamu, Hamidu Umar Pindiga, Rasheed Ajani Bakare, Abimbola Olu Osoba

**Affiliations:** Department of Medical Microbiology and Immunology, Federal Teaching Hospital Gombe, Gombe, Gombe state Nigeria; Department of Medical Microbiology and Parasitology, University College Hospital Ibadan, Ibadan, Oyo state Nigeria; Department of Histopathology, Federal Teaching Hospital Gombe, Gombe, Gombe state Nigeria; Department of Obstetrics and Gynaecology, Federal Teaching Hospital Gombe, Gombe, Gombe state Nigeria; Department of Radiotherapy and Oncology, Federal Teaching Hospital Gombe, Gombe, Gombe state Nigeria

**Keywords:** Human Papillomavirus, Genotypes, Cervix, Women, Nigeria

## Abstract

**Background:**

Sub-Saharan countries including Nigeria have the highest burden of Human Papillomavirus (HPV) infection in the world. Most studies on HPV surveillance in Nigeria were done in the southern part of the country. Geographical and socio-cultural diversity of Nigeria makes these data unlikely to be universally representative for the entire country. Northern Nigeria especially the North-East carries a higher prevalence of cervical cancer and many of its risk factors. The region may be harbouring a higher prevalence of HPV infection with a possibility of different genotypic distribution. This study was carried out to determine the burden and confirm the predominant HPV genotypes among women presenting for cervical cancer screening at the Federal Teaching Hospital Gombe (FTHG), North-eastern, Nigeria.

**Methods:**

The study was an observational hospital based cross sectional study among women who presented for cervical cancer screening in FTHG. A total of 209 consenting women were tested for cervical HPV infection using PCR. DNA sequencing was carried out on positive samples to determine the prevalent HPV genotypes.

**Results:**

The prevalence of cervical HPV infection among the participants with mean age of 39.6 ± 10.4 years was 48.1 %. The five most predominant genotypes were 18, 16, 33, 31 and 35, with prevalence of 44.7 %, 13.2 %, 7.9 %, 5.3 % and 5.3 % respectively. Other genotypes observed were 38, 45, 56, 58, 82 and KC5. Multiple HPV infections were detected among 7.9 % of participants. Risk factors such as level of education (*X*^2^ = 15.897; *p* = 0.007), age at sexual debut (*X*^2^ = 6.916; *p* = 0.009), parity (*X*^2^ = 23.767; *p* = 0.000), number of life time sexual partners (*X*^2^ = 7.805; *p* = 0.005), age at first pregnancy (*X*^2^ = 10.554; *p* = 0.005) and history of other malignancies (*X*^2^ = 7.325; *p* = 0.007) were found to have a statistically significant association with HPV infection.

**Conclusion:**

This study identified a high burden of HPV infection in Northern Nigeria while also confirming HPV 18 and 16 as the most predominant genotypes. It further justifies the potential benefit of the currently available HPV vaccines in the area. A larger and community based study is however recommended for better representation of the area.

**Electronic supplementary material:**

The online version of this article (doi:10.1186/s13027-015-0035-8) contains supplementary material, which is available to authorized users.

## Introduction

Among the approximately 200 known HPV types, about 40 can infect the cervix out of which 13 types (16, 18, 31, 33, 35, 39, 45, 51, 52, 56, 58, 59 and 66) from among the alpha species have been classified as group 1 carcinogens [1–3]. Of these oncogenic viruses, HPV-16 and HPV-18 are the most prevalent worldwide. They have been associated with about 70 % of invasive cervical cancer (ICC) worldwide and about 50 % of cervical intraepithelial neoplasia grade 3 (CIN3) [1, 4]. Aetiologically, Five HPV types (16, 18, 31, 33, and 45) have been linked to 80 % of cervical cancers while the other types contribute the remaining 20 % [5]. The high-risk HPV (hr-HPV) types are also implicated in vulvar, vaginal, penile, anal, and oropharyngeal neoplasms [5]. Infection of the genital tract with HPV is considered the most common sexually transmitted infection worldwide [5]. Several HPV genotypes tend to be transmitted together, leading to a high proportion (20–30 %) of concurrent infections with different types [1].

Worldwide the incidence and prevalence of HPV peaks at younger age soon after the start of sexual activity below the age of 25–35 years but subsequently declines with infections clearing as the infected individual grows older [5, 6]. Globally, age-adjusted prevalence of HPV in women with normal cytology is estimated at 10.4 %, ranging from 8.1 % in Europe to 22.1 % in Africa [5].

Persistent HPV infection of the cervical epithelium is the major risk factor for cervical cancer as more than 99 % of cases contain hr-HPV. Early/polygamous marriages [7], high parity, tobacco smoking, long-term hormonal contraceptive use, co-infection with *Chlamydia trachomatis*, *herpes simplex* virus type 2, HIV, immunosuppression, certain dietary deficiencies, genetic and immunological host factors have been observed to be major contributory factors to cervical cancer [8]. Other risk factors for cervical cancer include imbalanced vaginal flora, having an uncircumcised male partner and low socio-economic status [5, 9].

Cervical cancer is the second most common cancer in women worldwide and a leading cause of cancer deaths in developing countries [7]. The prevalence of cervical infection with HPV particularly high risk types that cause cervical cancer varies greatly worldwide [10].

Despite being among the areas with highest prevalence in the world, awareness about HPV and its association with cervical cancer is still poor in Nigeria [11]. The North-East zone of Nigeria has the highest poverty rates in the country [12]. Socio-cultural practices in the region also encourages early marriage and polygamy [13]. Despite cervical cancer being the commonest genital tract malignancy in this region (70.5 %), only 11.5 % of women ever had a Pap smear with very poor knowledge of HPV vaccine even among healthcare workers [14, 15].

In this study we determined the prevalence of HPV and its associated risk factors among women presenting for cervical cancer screening in Gombe, North-eastern Nigeria.

## Materials and methods

### Study area and population

The study was carried out among 209 women who presented for cervical cancer screening between August 2013 and May 2014 at the FTH Gombe. However, the screening for many of the participants was based on referral following risk assessment by healthcare staff. Ethical approval was obtained from Research and Ethics Committee of FTH Gombe. Recruited women were counselled and all those who gave informed consent were enrolled into the study.

### Specimen collection and storage

Rovers® Cervex-Brush® cell sampling device (Rovers Medical Devices B.V 5347 KV Oss, The Netherlands) was used for specimen collection. Specimen was obtained by inserting the cytobrush into the cervical canal and rotating it four times in a clockwise direction to collect all the cervical epithelial cells which adhered to the flat sides of the bristles.

The brush was then inserted into the vial containing preservative fluid. Liquid-based cytology system (*Liqui-PREP by* LGM International, Inc, Melbourne, FL, USA) for collection and transport of cervical specimen was used.

### DNA extraction

DNA from exfoliated cervical cells collected in the preservative fluid (Liqui-PREP) was extracted using proteinase K digestion, followed by phenol-chloroform extraction and ethanol precipitation. Gel extraction prior to DNA cleaning/purification for sequencing was done using QIAquick Gel Extraction Kit (QIAGEN Sample & Assay Technologies, Germany).

### Detection and typing of HPV

All the cervical specimens were tested for the presence of oncogenic HPV DNA using a nested Polymerase Chain Reaction (nPCR) with GP5+/GP6+ (GP5+ [5′ TTTGTTACTGTGGTAGATACTAC-3′] and GP6+ [5′- GAAAAATAAACTGTAAATCATATTC-3′]) [16] and PGMY 09/11 [17] consensus primers which amplifies a 150 bp fragment of the L1 HPV genomic region. AccuPower HotStart Premix (Bioneer Corporation, South Korea) was used for the PCR. Genotypic identification was achieved by direct sequencing using the Gp 6+ oligoprimer. Thermal cycler (Bio Rad) and Sequencing machine (Beckman Coultier CEQ 2000XL) were used for PCR and sequencing respectively. Sequence alignments were performed against various standard HPV genotype sequences stored in the GenBank database by on-line BLAST analysis to arrive at specific genotyping. The procedures were carried at the DNA Labs, Kaduna, Nigeria.

### Data analysis

Descriptive and inferential statistical analysis of the data was done using Statistical Package for the Social Sciences version 22 (SPSS Inc., Illinios, USA). Student’s *t* test was used to compare means for continuous variables, while categorical variables were summarized as proportions and further analyzed using Chi square to assess association between them. A logistic regression was performed to ascertain the effects of variables which showed a statistically significant association with HPV testing based on Chi square. Figures and tables were appropriately used in representing the analyzed data (Additonal file 1).

## Results

### Socio-demographic characteristics and HPV infection

Table 1 showed the association between socio-demographic characteristics of the participants and HPV infection. A total of two hundred and nine women participated in the study with their mean age being 39.6 years and standard deviation (SD) of ±10.4 years. Majority 127 (61.2 %) were between the ages of 30 and 45 years with only 36 (17.2 %) and 45 (21.5 %) being below 30 years and above 45 years respectively. The mean age of women found to be positive for HPV was 40.79 (±11.23) years compared to 38.49 (±9.47) years among those with no HPV infection. Student *t* test showed no statistically significant difference between the mean ages (*P* = 0.111).

**Table 1:** Association between socio-demographic factors and HPV infection

	Presence of HPV DNA		Total	*X* ^*2*^	*P* value
Age group (years)	Positive *N* (%)	Negative *N* (%)			
<30	17 (17.0 %)	19 (17.6 %)	036	0.642	0.725
30–45	59 (59.0 %)	68 (63.0 %)	127		
>45	24 (24.0 %)	21 (19.4 %)	045		
Total	100 (100 %)	108 (100 %)	208		
Type of Family					
Monogamous	59 (61.5 %)	69 (67.6 %)	128	0.829	0.363
Polygamous	37 (38.5 %)	33 (32.4 %)	070		
Total	96 (100 %)	102 (100 %)	198		
Level of Education					
No formal education	15 (15.0 %)	17 (15.9 %)	0 32	15.897	0.007*
Primary	10 (10.0 %)	04 (03.7 %)	014		
Secondary	24 (24.0 %)	27 (25.2 %)	051		
OND/NCE/HND	40 (40.0 %)	33 (30.8 %)	0 73		
University graduate	05 (05.0 %)	23 (21.5 %)	0 28		
Others	06 (06.0 %)	03 (02.8 %)	0 09		
Total	100 (100 %)	107 (100 %)	207		
Marital Status					
Married	88 (88.0 %)	94 (87.9 %)	182	1.963	0.580
Single	03 (03.0 %)	05 (04.7 %)	008		
Divorced	03 (03.0 %)	05 (04.7 %)	008		
Widow	06 (06.0 %)	03 (02.8 %)	009		
Total	100 (100 %)	107 (100 %)	207		
Employment Status					
Employed	47 (47.0 %)	52 (48.6 %)	099	0.053	0.818
Unemployed	53 (53.0 %)	55 (51.4 %)	108		
Total	100 (100 %)	107 (100 %)	207		
Religion					
Islam	53 (44.9 %)	65 (60.2 %)	118	1.092	0.296
Christianity	47 (52.2 %)	43 (38.8 %)	090		
Total	100 (100 %)	108 (100 %)	208		
Residence					
Within Gombe city	75 (75.0 %)	87 (81.3 %)	162	1.260	0.533
Other LGAs	18 (18.0 %)	15 (14.0 %)	033		
Outside Gombe state	07 (07.0 %)	05 (04.7 %)	012		
Total	100 (100 %)	107 (100 %)	207		
Tribe					
Fulani	28 (28.0 %)	29 (27.1 %)	057	3.345	0.341
Tangale	26 (26.0 %)	18 (16.8 %)	044		
Hausa	11 (11.0 %)	17 (15.9 %)	028		
Others	35 (35.0 %)	43 (40.2 %)	078		
Total	100 (100 %)	107 (100 %)	207		
Type of housing					
Personal house	50 (50.0 %)	63 (58.9 %)	113	6.023	0.197
Rented flats	27 (27.0 %)	32 (29.9 %)	059		
Self contained	20 (20.0 %)	10 (09.3 %)	030		
Single rooms	02 (02.0 %)	02 (01.9 %)	004		
Others	01 (01.0 %)	00 (00.0 %)	001		
Total	100 (100 %)	107 (100 %)	207		

* = statistically significant (i.e. <0.05), OND (Ordinary National Diploma), HND (Higher National Diploma), NCE (National Certificate of Education), LGA (Local Government Area)

More than half, 110 (53.4 %) were educated up to tertiary level with only 32 (15.4 %) having no formal education while 14 (6.7 %) and 51 (24.5 %) had primary and secondary education respectively. Nearly all, 182 (88.0 %) were married as both single and divorced women accounted for only 3.8 % each while 4.3 % were widows. Majority, 128 (64.8 %) of the women were from monogamous family settings. Less than half, 99 (48.1 %) were employed (government and private) with all the remaining 108 (51.9 %) being unemployed among which house wives were predominant. Muslims were slightly more 118 (56.5 %) than Christians 90 (43.5 %).

Except for level of education, other socio-demographic characteristics did not show any statistically significant association with HPV infection.

### Analysis of HPV infection

**Fig. 1:**
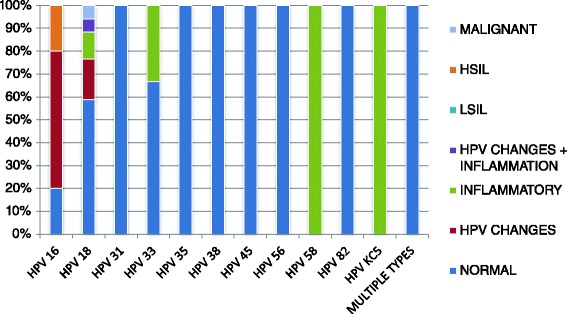
Percentage distribution of different cytological findings in comparison with corresponding HPV types. HPV types 31, 35, 38, 45, 56, 82 and multiple infections were only present in women with normal cytological findings. HPV 58 and KC5 were only found among women with inflammatory cytological findings. HPV 16 had 20 % normal, 60 % HPV changes and 20 % HSIL. HPV 18 had 58.8 % normal, 17.6 % HPV changes, 11.8 % inflammatory, 5.9 % malignant and 5.9 % HPV changes with inflammation. HPV 33 had 66.6 % normal and 33.3 % inflammatory

HPV DNA was detected in 100 (48.1 %) of the participants while 108 (51.9 %) were negative for the virus. Overall, ten different HPV types were identified from this study. HPV types 18 and 16 were the most predominant with proportions of 44.7 % and 13.2 % respectively. Multiple HPV infections were identified among 7.9 % of the specimens. Other genotypes identified were types 31, 33, 35, 38, 45, 56, 58, 82 and KC5 with corresponding proportions of 5.3 %, 7.9 %, 2.6 %, 2.6 %, 2.6 %, 2.6 %, 2.6 %, 5.3 % and 2.6 % respectively. Of the 209 participants, normal cytological findings were observed among 126 (61.6 %) while 80 (39.0 %) had abnormal features and 3 (1.4 %) had unsatisfactory smears for cytologic studies. Figure 1 shows the percentage distribution of the different cytological findings in comparison with corresponding HPV types.

### Risk factors for HPV infection

Table 2 showed the association between HPV infection and some risk factors within the participants. Of the 67 participants who had early sexual debut (earlier than 18 years of age), 41 (61.2 %) were positive for HPV DNA as against 57 (41.6 %) of the respondents who started their sexual life at or later than 18 years of age. Early sexual debut showed a statistically significant association with HPV infection (*X*^2^ = 6.916, *P* = 0.009). Multiple life time sexual partners also conferred a significant risk for HPV infection (*X*^2^ = 7.805, *P* = 0.005) as 26 (68.4 %) out of the 38 respondents with multiple sexual partners were HPV positive compared to 71 (43.3 %) out of 164 with single life time sexual partner. Statistically significant association was also observed between ages at first pregnancy (primigravidity) and HPV infection (*X*^2^ = 10.554, *P* = 0.005) as respondents with history of early pregnancy (less than 18 years) showed higher percentage of HPV infection (75.0 %) in comparison to 43.7 % and 42.9 % for normal and late age of primigravidity respectively. Parity of the participants also showed a statistically significant association with HPV infection (*X*^2^ = 23.767, *P* = <0.001) with grand multiparous and great grand multiparous women showing a higher percentage of infection; 62.3 % and 80 % respectively. Although women in a polygamous family setting showed higher proportion of HPV infection than those in monogamous setting, the association was not statistically significant (*X*^2^ = 1.517, *P* = 0.468). Family history of cervical cancer did not reveal a significant association with HPV infection (*X*^2^ = 0.719, *P* = 0.397) but history of other malignancies had a statistically significant association with HPV infection (*X*^2^ = 7.325, *P* = 0.007). Among those with history of other malignancies, the type of malignancy (gynaecologic or others) did not show a statistically significant association with HPV infection (*X*^2^ = 0.522, *P* = 0.470).

**Table 2:** Association between some risk factors and HPV infection

Variable	Presence of HPV DNA		Total	*X* ^*2*^	*P* value
Age at sexual debut	Positive	Negative			
	*N* (%)	*N* (%)			
<18 years	41 (61.2 %)	26 (38.8 %)	67 (100 %)	6.916	0.009*
≥18 years	57 (41.6 %)	80 (58.4 %)	137 (100 %)		
Lifetime sexual partners					
Single	71 (43.3 %)	93 (56.7 %)	164 (100 %)	7.805	0.005*
Multiple	26 (68.4 %)	12 (31.6 %)	38 (100 %)		
Age at primigravidity					
<18 years	24 (75.0 %)	08 (25.0 %)	32 (100 %)	10.554	0.005*
18–28 years	69 (43.7 %)	89 (56.3 %)	158 (100 %)		
>28 years	03 (42.9 %)	04 (57.1 %)	07 (100 %)		
Parity					
Nulliparous	06 (54.5 %)	05 (45.5 %)	11 (100 %)	23.767	<0.001*
Primiparous	02 (15.4 %)	11 (84.6 %)	13 (100 %)		
Multiparous	32 (35.6 %)	58 (64.4 %)	90 (100 %)		
Grand multiparous	48 (62.3 %)	29 (37.7 %)	77 (100 %)		
Great-grand multiparous	12 (80.0 %)	03 (20.0 %)	15 (100 %)		
Presence of co-wives					
No other wife	59 (46.1 %)	69 (53.9.7 %)	128 (100 %)	1.517	0.468
One other wife	20 (50.0 %)	20 (50.0 %)	40 (100 %)		
≥2 other wives	17 (58.6 %)	12 (41.4 %)	29 (100 %)		
Family history of cervical cancer					
No	85 (47.0 %)	96 (53.0 %)	181 (100 %)	0.719	0.397
Yes	14 (56.0 %)	11 (44.9 %)	25 (100 %)		
History of other malignancies					
No	80 (44.7 %)	99 (55.3 %)	53 (100 %)	7.325	0.007*
Yes	19 (73.1 %)	07 (26.9 %)	26 (100 %)		
Type of other malignancies					
Gynaecological	17 (73.9 %)	06 (26.1 %)	23 (100 %)	0.522	0.470
Others	01 (50.0 %)	01 (50.0 %)	02 (100 %)		
HIV Status					
Positive	07 (63.6 %)	04 (36.4 %)	11 (100 %)		0.222**
Negative	47 (43.5 %)	61 (56.5 %)	108 (100 %)		
Hormonal contraceptives					
No	50 (45.0 %)	61 (55.0 %)	111 (100 %)	0.874	0.350
Yes	48 (51.6 %)	45 (48.4 %)	93 (100 %)		
Use of condom					
No	73 (50.3 %)	72 (49.7 %)	145 (100 %)	1.026	0.311
Yes	26 (42.6 %)	35 (57.4 %)	61 (100 %)		

** = Fischer’s exact test

* = statistically significant (i.e. <0.05)

Other factors as participants’ HIV status, partners’ use of condom and use of hormonal contraceptives did not show statistically significant association with HPV infection. Although not shown in any of the tables, history suggestive of genital tract infection (vaginal discharge, dyspareunia and vaginal bleeding), history of alcohol consumption and cigarette smoking did not show statistical significance in associated with HPV infection. Partners’ circumcision status could not be analyzed as all respondents had circumcised sexual partners.

Logistic regression was performed to ascertain the effects of level of education, age at sexual debut, parity, number of sexual partners age at primigravidity and history of other malignancies on the likelihood that participants have HPV infection (Table 3).

**Table 3:** Logistic regression analysis of some risk factors for HPV infection

	Regression Coefficient	*P*-value	Odds ratio	95 % CI
Level of education				
No formal education (REF)			1.000	
Primary	0.818	0.301	2.267	0.481–10.680
Secondary	−0.223	0.809	0.800	0.131–4.874
OND/NCE/HND	0.811	0.286	2.250	0.507–9.993
University graduate	2.219	0.010*	9.200	1.698–49.858
Others	0.501	0.502	1.650	0.383–7.109
Age at sexual debut				
≥18 years (REF)			1.000	
<18 years	−0.794	0.009*	0.452	0.249–0.821
Parity				
Nulliparous (REF)			1.000	
primiparous	1.204	0.174	3.333	0.588–18.891
Multiparous	3.091	0.002*	22.000	3.076–157.341
Grandmultiparous	1.981	0.004*	7.250	1.905–27.598
Great-grand multiparous	0.882	0.199	2.417	0.629–9.290
Sexual partner (s)				
Single (REF)			1.000	
Multiple	1.043	0.006*	2.838	1.340–6.011
Age at primigravidity				
18–28 years (REF)			1.000	
<18 years	−1.386	0.109	0.250	0.046–1.365
>28 years	−0.033	0.966	0.967	0.210–4.466
History of other malignancies				
No (REF)			1.000	
Yes	0.363	0.398	1.437	0.619–3.336

* = Statistically significant (i.e. <0.05)

*REF* Reference Category, *OND* Ordinary National Diploma, *HND* Higher National Diploma, *NCE* National Certificate of Education

## Discussion

This study reveals a 48.1 % prevalence of cervical HPV infection among women who presented for cervical cancer screening in Gombe, north-eastern Nigeria. It is one of the highest prevalence rates among one of the oldest studied populations (mean age of 39.6 ± 10.4 years) to be ever reported from any part of Nigeria and the first from North-Eastern Nigeria [6, 10, 11, 18–20]. Although there are only a few studies from Northern Nigeria, a high prevalence of 76 % reported from Kano further highlights the possibility of this region carrying the highest HPV burden in the country [18].

Several Nigerian studies among diverse population groups had reported lower prevalence rates including 19.6 % among HIV infected patients in Lagos [19], 26.3 % in Ibadan [11], 14.7 % in Irun [6] and 25 % in Abuja [21]. This disparity may be explained by variations among the different study populations with varying exposures to different risk factors based on diverse socio-cultural and geographical differences. This study was conducted among women presenting for cervical cancer screening with many of them being referred by a healthcare worker following complaints of symptoms suggestive of either genital tract infection or cervical cancer. Hence, they are naturally a selected “high risk” group for HPV infection. There is widespread polygamy and early marriage [13], with cervical cancer being the commonest genital tract malignancy in this poorest region of the country [12, 14]. Early pregnancy and high parity are all very common in North-Eastern Nigeria [22]. These and probably other unreported risk factors in the locality could be responsible for the high prevalence of HPV infection in this “high risk region”.

Studies done in other parts of sub-Saharan Africa among similar population groups have shown similarly high prevalence rates when compared to the value in this study. A prevalence of 66.1 % had been reported among high risk women in Burkina faso [23] while 49.6 % and 60.7 % were also reported among women in Kenya and Sudan respectively [24, 25]. In contrast, some non-African countries showed lower prevalence even among similar population groups with 5.5 % in Iran [26], and 23.6 % in China [27]. This has further buttressed the fact that sub-Saharan Africa, particularly Nigeria carries one of the highest burden of HPV infection in the world [7, 11]. The predominance of most positive risk factors for HPV alongside inadequate knowledge and awareness about this virus in Africa could be an explanation for the higher prevalence of the infection in this continent. Moreover, from evolutionary point of view, there are predictions that the root of some if not all HPV types could be traced to Africa [28].

The predominance of HPV types 16 and 18 as observed in this study follows the global trend and is in conformity with some Nigerian studies [11, 18]. However, the observed predominance of HPV 18 as against type 16 in this study is in concert with a similar study from Kano in Northern Nigeria [18]. This suggests the possibility of Northern Nigeria harbouring a higher prevalence of HPV 18 than 16 in contrast to the general global trend. Although very few studies are available to justify this assertion, geographical differences associated with HPV type distribution had been previously reported from other parts of the world [29]. Variations were particularly observed in predominance between the other HPV types found in this study in comparison to most other Nigerian studies from southern part of the country [10, 11, 19]. The predominant HPV types were also different from those reported in Abuja (North-central Nigeria) [21]. This may be attributable to geographical variations in addition to culture and other life styles or partly because most of the studies restricted their search to specific HPV types while this study had the advantage of using DNA sequencing to detect wider variety.

Seven (HPV types 16, 18, 31, 33, 35, 45 & 58) out of the eight (HPV types 16, 18, 31, 33, 35, 45, 52 and 58) global most common cervical HPV types among women were also found in this study [30]. The five HPV types (16, 18, 31, 33 and 45) responsible for 80 % of cancers [31] are also about the most predominant in this study accounting for 13.2 %, 44.7 %, 5.3 %, 7.9 % and 2.6 % for HPVs 16, 18, 31, 33 and 45 respectively. This may probably have contributed to the higher prevalence of cervical cancer in Northern Nigeria [14]. HPV- KC5 has been reported to be present in the normal skin among some rural dwellers in China [32]. More recently, it was also found in the respiratory tract suggesting the possibility of its presence in some other parts of the body [33]. HPV-KC5 may not confer a risk for cervical cancer as it does not belong to alpha HPV species which contain all the types responsible for cervical cancer [3].

Low prevalence of multiple HPV infections as observed in this study compared to studies that used type specific primers (TS-PCR) in genotyping is not unusual [18]. Sequencing has however been shown to detect more genotypes than TS-PCR suggesting the need to combine both methods for maximum yield [34]. This study utilized nested PCR using PGMY 09/11 and GP5+/GP6+ which has been shown to be sensitive in detecting wider range of HPV genotypes and also relatively more effective in detecting multiple infections[35]. However, the inability of this study to detect low risk HPV genotypes may be attributable the PGMY 09/11 primer which has low capacity of detecting lr-HPV and the fact they are generally fewer in cervical specimens and also more common among males than females [35–37].

Several variables (level of education, age at sexual debut, age at first pregnancy, parity, lifetime sexual partners and history of other malignancies) observed in this study revealed a statistically significant association with HPV infection. Low socio-economic status, early marriage/coitarche, polygamy and high parity are all well-established risk factors for HPV infection [7, 38]. Women with lower levels of education were found to have a higher risk of HPV infection as shown in this study and another Nigerian study [21]. Higher educational level among women is generally associated with increased knowledge/attitude towards HPV and its preventive measures with minimal risk factors for the infection[39]. This has also been observed in this study as many of participants were highly educated up to tertiary level as against the corresponding 2 % among the general female population in the area[12]. Therefore, this study may not depict the true picture of the general population. A study among cervical cancer screening population group in Lagos, Nigeria also reaffirmed the statistical significance of age at sexual debut, parity and lifetime sexual partners as risk factors for HPV infection [20]. In contrast to this study, Adegbesan-Omilabu et al., and Rocha-Brischiliari et al. all reported the use of oral contraceptive pills, HIV infection and cigarette smoking as significant risk factors for HPV infection [2, 40]. This may be attributed to the relatively few percentage of women that responded to questions on smoking history (0.5 %), use of hormonal contraception (44.5 %) and HIV infection (5.2 %). Possible variations in the duration of exposure to these factors among different studied populations may also be contributory. Women with longer duration of exposure to oral contraceptives have been shown to have 2-fold increase in risk of acquiring HPV infection [41]. In concert with this study, marital status, presence of co-wives, religion and tribe were all not statistically significant risk factors for HPV as highlighted by some Nigerian studies [20]. Also unrelated to the risk of HPV infection in this study is the use of condom as has also been observed in Ibadan by Thomas et al. [10].

Majority of the studied population fell within the age group 30 to 45 years, hence are suitable for enrolment in screening and treatment programmes in preventing cervical cancer [6]. In addition, up to 166 (79.8 %) of the participants in this study were above 30 years of age which shows that majority may be having a persistent HPV infection (the actual risk for developing cervical cancer). This finding further reaffirms the suitability of HPV DNA detection as an important diagnostic tool for cervical cancer screening in this population [42].

Logistic regression analysis of the identified risk factors showed that university education, multiple sexual partners, multiparity and grand multiparity are associated with a significantly increased likelihood of having HPV infection among the participants. The apparent significance of university education as a risk factor against our earlier observation and the general trend may not be unconnected with skewed distribution of the respondents in terms of educational qualification. These findings further confirm the need for a larger and community based study to obviate the lopsided distribution of the participants as majority of them are educated and screening is mostly based on referral by healthcare workers.

## Conclusion

HPV Prevalence of 48.1 % was recorded from this study. Eleven different HPV types were detected with the five most predominant types (sequentially) being 18, 16, 33, 31 and 35. Other types were 38, 45, 56, 58, 82 and KC5. Low level of education, early age at sexual debut, high parity, multiple life time sexual partners, early age at first pregnancy and positive history of other malignancies were the significant risk factors associated with cervical HPV infection in Gombe.

This study has revealed north-eastern Nigeria as having one of the highest HPV prevalence in the country and also a region that will benefit from the currently available HPV vaccines.

There is need for a universal, well-coordinated, multi-centred, rural and urban community based study on HPV in addition to a comprehensive national cervical cancer screening programme and improved availability/accessibility of the currently available HPV vaccines in all parts of Nigeria.

## Additional file

Additional file 1:
**Detailed Methodology.** (DOCX 21 kb)

## Abbreviations

CI: Confidence interval
CIN: Cervical intraepithelial neoplasia
FTHG: Federal Teaching Hospital Gombe
HPV: Human papillomavirus
hr: High risk
lr: Low risk
ICC: Invasive cervical cancer
OR: Odds ratio
PCR: Polymerase chain reaction
