# Supplementary material for: Epidemiological patterns of cervical human papillomavirus infection among women presenting for cervical cancer screening in North-Eastern Nigeria
Source: Infect Agent Cancer. 2015 Oct 2;10:39. doi: 10.1186/s13027-015-0035-8 (PMC4592568; doi:10.1186/s13027-015-0035-8)
Supplement: Additional file 1: — Detailed Methodology. (DOCX 21 kb) [file 13027_2015_35_MOESM1_ESM.docx]

**SAMPLE COLLECTION AND STORAGE**

Rovers® Cervex-Brush® cell sampling device (Rovers Medical Devices B.V 5347 KV Oss, The Netherlands) was used for the Liquid-based cytology. It was inserted into the cervical canal and rotated five times in a clockwise direction whilst applying light pressure to collect all the cervical epithelial cells which adhered to flat sides of the bristles. The brush was then inserted into a vial with preservative fluid. This procedure was carried out by Doctors in the department of Obstetrics and Gynaecology of the federal teaching hospital Gombe.

Liquid-based Cytology System (*Liqui-PREP by* LGM International, Inc, Melbourne, FL, USA) which utilizes a preservative for collection and transport of cervical specimen was used. It is specifically designed to be molecular & immunochemistry friendly for follow-up testing. Also, it is lytic for bloody specimens, digests mucus and has anti-bacterial effect to suppress growth while in transport. It preserves the specimen integrity for up to 90 days at room temperature.

**LABORATORY METHODS**

All the cervical specimens were tested for the presence of oncogenic HPV DNA using a nested Polymerase Chain Reaction (nPCR) with GP5+/GP6+ (GP5+ [5' TTTGTTACTGTGGTAGATACTAC-3'] and GP6+ [5'- GAAAAATAAACTGTAAATCATATTC-3']) and PGMY 09/11[15] consensus primers which amplifies a 150bp fragment of the L1 HPV genomic region. AccuPower HotStart Premix (Bioneer Corporation, South Korea) was used for the PCR. Genotypic identification was achieved by direct sequencing using the Gp 6+ oligoprimer. Staining of slides for cytology and their subsequent reading and reporting was done in the department of Histopathology, FTH Gombe.

**DNA EXTRACTION**

From the LBC cervical specimen, about 2 mls of the cell suspension was centrifuged at 10,000 rpm for ten minutes. The centrifugation step was repeated 3 times to obtain enough sediment. The sediment obtained was then resuspended in 400μl of detergent buffer (10mM Tris-HCl, 50mM KCl, 2.5mM MgCl_2_, 0.45% Triton X100, 0.45% Tween 20) and 100μg/ml (40μl) proteinase K, mixed and incubated at 55°C for 2hours. This was followed by the addition of 400μL of phenol chloroform, vortexing and further centrifugation at 10,000 rpm for 20 minutes. The upper layer was carefully pipetted and transferred to a new tube while leaving the middle (proteins and contaminants) and the lower (phenol chloroform) layers in the initial tube which was discarded. About 30μL of 3M Na acetate and 1ml of 100% ethanol were added, vortexed and then kept frozen overnight for DNA to precipitate. The contents were then centrifuged at 4°C (ALC high speed refrigerated centrifuge, Italy) for 1 hour before decanting the supernatant (while preserving the pellets) and addition of 200μl of 70% ethanol. This was further centrifuged at 14,000 rpm for 10 minutes at room temperature and the whole ethanol removed through vacuum drying. The precipitated DNA was re-suspended in 30μl of DNAse free water and stored in aliquots at -20°C until ready for PCR.

**MATERIALS AND REAGENTS USED**

Standard PCR reagents that were used included a set of GP5+/GP6+ consensus primers (GP5+ [5'- TTTGTTACTGTGGTAGATACTAC-3'] and GP6+ [5'-GAAAAATAAACTGTAAATCATATTC-3']) and PGMY primers. Also used are TAE buffer and distilled water. Others are AccuPower HotStart Premix (Bioneer Corporation, South Korea) which provides 1mM total concentration of dNTP, Bioneer’s HotStart DNA polymerase, pyrophosphatase, pyrophosphate, PCR buffer with 1.5mM MgCl_2_, stabilizer and tracking dye.

Materials included gloves, PCR tubes and caps, PCR tube racks, ethanol-resistant markers, and set of micropipettes that dispense between 1 - 10 μl (P10), 2 - 20 μl (P20), 20 - 200 μl (P200) and 200 - 1000 μl (P1000). Others include microcentrifuge, cold centrifuge, heating blocks, equipment for agarose gel electrophoresis and geldoc with a digital camera for visualization and documentation to an attached computer. Thermal cycler (Bio Rad) and Sequencing machine (Beckman Coultier CEQ 2000XL) were used for PCR and sequencing respectively.

**PROCEDURE FOR PCR**

**SETTING UP A REACTION MIXTURE**

Reagents that were to be added to the reaction mixture were assembled and arranged in the order that they were used. The PCR tubes were labeled with ethanol-resistant marker. A 20 μl reaction mixture was made with each of the AccuPower HotStart PCR premix containing 0.5μl of each of the 18 PGMY primers (5Picomole dilution) i.e. 9 μl, 2 μl of template DNA and 9μl of distilled water (dH_2_O).

| PGMY09/11 primers | 9 μl (0.5 μl X 18μl) |
| --- | --- |
| Template DNA | 2 μl |
| Distilled water | 9 μl |
| Total | 20 μl |

A second round PCR was set up by taking 1 μl of the first round PCR products in lieu of the template DNA and 2 μl of GP5+ (10μM), 2μl of GP6+ (10μM) and 15μl of distilled water (dH_2_O) to get the 20 μl reaction mixture as above.

**PCR PROTOCOLS**

The 96 well plate (premix) was be placed on ice pack while the reaction mixture was constituted as highlighted above. The reaction mixture was gently mixed on a microcentifuge before being placed into the thermal cyler.

The lid to the thermal cycler was firmly closed to start the program. Amplification cycles for the PGMY09/11 primers were set as follows; 94°C for 5 minutes, 94°C for 1 minute, 72°C for 1 minute and finally 72°C for 5 minutes in a 35 cycle reaction. In the second round PCR (using GP 5+/GP 6+ primers), the conditions were as follows; 5 minutes at 94°C (denaturation), 30 seconds at 94°C, 30 seconds at 38°C and 30seconds at 72°C, with the final extension step prolonged to 5 minutes at 72°C. A total of 35 cycles were set up.

**GEL ELECTROPHORESIS**

A 2% agarose gel was prepared by mixing and heating 2 grams of gel powder in 100mls of 2% TAE buffer solution. One litre of 2% buffer solution was made by adding 20mls of 50X buffer to 980mls of dH_2_O. Ethidium bromide (10μl) was added when the mixture was cold. Appropriate sized gel combs were placed on to a template container and the mixture poured before being allowed to solidify. The gel was carefully removed and placed in the electrophoresis tank containing 2% buffer solution. Twenty (20) μl of molecular weight marker (100bp X 1000) and 10μl of the PCR products were appropriately loaded in to the gel and electrophoresis was allowed to run at 90 volts for 90 minutes. The gel was transferred and viewed in the gel doc (Bio-Rad gel doc 2000, USA).

**INTERPRETATION**

If the PCR product in question is present, the ethidium bromide will intercalate between the bases of the DNA strands and appear as bands which will be visualized by UV-transillumination. Band sizes of interest were located at 150bp and compared with the molecular weight marker. Being a mutagen/carcinogen, ethidium bromide was cautiously handled and disposed appropriately. Nitrile gloves, eye protection and fully buttoned lab coats were used. Gels with ethidium bromide were handled and disposed as harzadous wastes.

**QUALITY CONTROL**

In separate PCR tubes, all the reagents with the exception of template DNA were added and this served as a negative control (water was increased to compensate for the missing volume). In another reaction which served as positive control, template DNA of HPV type 18 known to amplify under the same conditions as the experimental PCR was added. The positive and negative controls were part of the entire process up to gel electrophoresis.

**PROTOCOL FOR DNA EXTRACTION AND PURIFICATION FOR SEQUENCING**

From the agarose gel of nested PCR products, appropriate sized bands (bands of interest) were carefully cut out. Gel extraction was done using QIAquick Gel Extraction Kit (QIAGEN Sample & Assay Technologies, Germany). Sequencing PCR reaction mixture was set up with a final volume of 10μL i.e. 4μL DTCS Quick Start Master Mix, 1μL GP 6+ primer (0.3μM) and 5μL DNA template. Sequencing PCR reaction conditions were as follows; 90°C for 30 seconds, 80°C for 30 seconds and 60°C for 4 minutes; all for 30 cycles, followed by a hold at 4°C.

After the cycles were completed, the tubes were spin down to collect any condensate. The samples were then concentrated by ethanol precipitation.

Ethanol precipitation was achieved by transfering 10μl of the labeled PCR products to a microcentrifuge tube, and adding 2.5μl of freshly prepared clean-up mixture (containing 2μl of Na acetate (3M), 2μl of Na EDTA (0.5M) and 1μl glycerol). These were mixed and 30μl of ice-cold 95% ethanol was added to each sample. This was followed by brief vortex mixing and spinning at 12,000 rpm at 4°C for 15 minutes. The supernatant (ethanol) was then aspirated from the tube while ensuring stability of the pellet. The pellet was carefully washed by adding 100μl of cold 70% ethanol (stored at-20°C). The sample was carefully spun at 12,000 rpm at 4°C for 2 minutes without vortexing. Ethanol was completely removed and vacuum dried. The pellets were resuspended in 33μl sample loading solution (SLS) and can be stored for about a week at 4°C or for about a month at -20°C until ready for sequencing. DNA Sequencing was done using Beckman Coulter CEQ 2000XL sequencing machine.

Sequence alignments were performed against various standard HPV genotype sequences stored in the GenBank database by on-line BLAST analysis to arrive at specific genotyping.
